# Retinopathy of prematurity: Accuracy of ROPScore and WINROP algorithms in a Brazilian population

**DOI:** 10.5935/0004-2749.2024-0170

**Published:** 2024-12-26

**Authors:** Amanda F. L. Morais, Luisa M. Hopker, Nilva S. B. Moraes, Bernardo Reichert, Murilo V. De Prá, Anna Carolina B. Linhares, Ricardo M. Takashima, Norma Allemann

**Affiliations:** 1 Department of Ophthalmology and Visual Sciences, Escola Paulista de Medicina, Universidade Federal de São Paulo, São Paulo, SP, Brazil; 2 Department of Ophthalmology, Hospital de Olhos do Paraná, Curitiba, PR, Brazil; 3 Médicos de Olhos S.A., Curitiba, PR, Brazil; 4 Universidade Federal do Paraná, Curitiba, PR, Brazil; 5 Universidade Positivo, Curitiba, PR, Brazil

**Keywords:** Retinopathy of prematurity, ROPScore, WINROP, Prediction algorithm, Infant, premature

## Abstract

**Purpose:**

To assess the sensitivity and specificity of the retinopathy of prematurity score (ROPScore) and weight, insulin-like growth factor-1, retinopathy of prematurity algorithm in predicting the risk of developing severe retinopathy of prematurity (prethreshold type 1) in a sample of preterm infants in Brazil.

**Methods:**

Retrospective analysis of medical records of preterm infants (n=288) with birth weight of ≤1500 g and/or gestational age of 23-32 weeks in a neonatal unit in Southern Brazil from May 2013 to December 2020 (92 months).

**Results:**

The incidence of confirmed severe retinopathy of prematurity was 6.6%. ROPScore showed a 100% sensitivity, 44.6% specificity (95% confidence interval [CI] 38.7-50.6), 11.3% positive predictive value (95% CI 6.5-16.1), and 100% negative predictive value in predicting severe retinopathy of prematurity. The weight, insulin-like growth factor-1, retinopathy of prematurity algorithm demonstrated a 78.9% sensitivity (95% CI 60.6-97.3), 51.3% specificity (95% CI 45.3-57.3), 10.3% positive predictive value (95% CI 5.3-15.2), and 97.2% negative predictive value (95% CI 94.5-99.9).

**Conclusion:**

ROPScore identified all patients at risk for severe retinopathy of prematurity. These findings support incorporating ROPScore into Brazilian guidelines to optimize retinopathy of prematurity screening and reduce unnecessary ophthalmologic examinations. Weight, insulin-like growth factor-1, retinopathy of prematurity’s suboptimal performance in this Brazilian sample highlights the need for country-specific algorithm adjustments.

## INTRODUCTION

Retinopathy of prematurity (ROP) has far-reaching consequences, imposing significant financial and social burdens on communities. Beyond the risk of irreversible vision loss, ROP can also lead to cognitive and psychomotor impairments, impacting the long-term development of the affected children^(1^,^2)^. The current ROP screening process, involving ophthalmological examinations, can be distressing for premature infants^(3^,^4)^. Furthermore, there is a scarcity of experienced ophthalmologists for ROP screening in both high and low-income countries^(5)^. Therefore, it is imperative to assess the currently available screening algorithms to facilitate the detection of preterm newborns at risk of developing ROP and requiring treatment. This can help optimize the screening protocols, reducing the number of unnecessary examinations for low-risk children^(6^-^8)^.

The Weight, Insulin-like Growth Factor-1, Retinopathy of Prematurity (WINROP) algorithm, developed in Sweden, is a predictive tool to identify newborns at risk of severe ROP. This online application is designed for newborns with a gestational age (GA) between 23 and 32 weeks. The algorithm functions by comparing the newborn’s weight each week with a normalized growth curve for infants who did not develop ROP or who developed mild ROP. Any differences between the expected and actual weights accumulate each week. When these cumulative deviations exceed a predetermined threshold, the system triggers a red alert, signaling the risk of development of severe ROP development in the newborn^(9^-^11)^.

The Retinopathy of Prematurity Score (ROPScore) algorithm was developed in Brazil to predict severe ROP. It utilizes birth weight (BW), GA, weight gain proportional to body weight at 6 weeks of life, need for blood transfusion, and use of oxygen in mechanical ventilation as predictive variables. The algorithm’s creator proposed that ROPScore evaluation can be performed in the 2^nd^ week of life instead of the 6^th^ week, allowing for earlier screening^(12)^. A score of ≥11 indicates a risk of ROP (any stage), while a score of ≥14.5 signals a risk of severe ROP^(7)^. Infants with ROP score of ≥14 require more frequent monitoring owing to the high risk of developing severe ROP.

The primary objective of this study was to evaluate the sensitivity, specificity, positive predictive value (PPV), and negative predictive value (NPV) of ROPScore and WINROP for predicting the risk of developing ROP or severe ROP (prethreshold type 1), as well as the accuracy of these algorithms.

## METHODS

### Study design and participants

This observational, cross-sectional, retrospective study analyzed data from the neonatal intensive care unit at Hospital do Trabalhador in Brazil, covering a 92-month period from May 2013 to December 2020. The inclusion criteria was newborns with a BW of ≤1,500 kg and/or GA of 23-32 weeks who underwent ROP screening and for whom the necessary medical data for the application of ROPScore and WINROP were available.

Out of the 321 premature infants reviewed, 6 were excluded due to incomplete medical records, and 27 were excluded because the GA exceeded 32 weeks. Therefore, 288 premature infants were included in the analysis.

### ROP screening and classification

All premature infants enrolled in this study underwent ophthalmologic examinations performed by a single ophthalmologist between the 4^th^ and 6^th^ week of life. The examinations adhered to the Brazilian guidelines, continuing up to the GA of 45 weeks, until complete retinal vascularization or complete regression of ROP^(13)^. Examination frequency varied, occurring weekly or less frequently, contingent upon the ophthalmological findings. Before the examination, pupils were dilated using three instillations spaced five minutes apart. A drop of 0.5% tropicamide (Mydriacyl 0.5%^®^, Alcon Laboratórios do Brasil Ltda.) and a drop of 2.5% phenylephrine hydrochloride (Fenilefrina 2.5%^®^, Allergan Produtos Farmacêuticos Ltda.) were used approximately 40 minutes before the examination. Retinal fundus examination was then performed using a binocular indirect ophthalmoscope and a 28-diopter lens. The premature infants were positioned in dorsal decubitus. A blepharostat was used after the instillation of anesthetic eye drops.

Severe ROP was defined as ROP requiring treatment (type 1 prethreshold ROP), in accordance with the early treatment for retinopathy of prematurity (ETROP) criteria^(14)^.

### WINROP algorithm

The algorithm is available online (www.winrop.com). On the website’s homepage, a unique identifier was created for each newborn, and their date of birth, estimated due date (GA of 40 weeks), GA, and BW were inputted. Subsequently, the weekly weights of each premature infant, obtained from their electronic medical records, were added. These weekly weights were included until either the algorithm triggered an alarm signal or the infant was discharged. The platform then indicated whether a red alarm signal was triggered, signifying a risk of developing severe ROP, along with the specific week the signal was activated. Subsequently, newborns were divided into two groups based on the presence or absence of the WINROP alarm signal. The online model’s performance was then evaluated by calculating the sensitivity (probability of red alarm signal given confirmed severe ROP) and specificity (probability of no red alarm signal given no severe ROP). Using these values, along with the 6.6% prevalence of confirmed severe ROP (19/288), the PPV and NPV were calculated. The PPV indicated the probability of confirmed severe ROP given a positive red alarm signal. The NPV indicated the probability of not having severe ROP given a negative red alarm signal. Additionally, the overall accuracy of the WINROP algorithm was calculated, reflecting the probability of correct predictions.

### ROPScore algorithm

The ROPScore algorithm was applied using the smartphone application “ROP SCORE 3” for IOS (PABEX Corporation). The following data were entered into the application: BW, GA, whether a blood transfusion occurred in the first 6 weeks of life, oxygen use through mechanical ventilation in the first 6 weeks of life, and weight at two weeks of life. The application then calculated the ROPScore based on these inputs. The ROP Score’s performance was also evaluated by calculating sensitivity (the probability of obtaining an ROP Score of ≥11 or ≥14.5 given that the newborn has confirmed ROP [any stage] or confirmed severe ROP, respectively) and specificity (the probability of scoring below these thresholds [<11 for any ROP stage and <14.5 for severe ROP] when ROP was not confirmed). Finally, the PPV and NPV were calculated for both ROP and severe ROP using the previously determined sensitivities and specificities. These calculations incorporated the study’s observed prevalence of confirmed ROP, which was 38.2% (110/288). The PPV indicated the probability of confirmed ROP (any stage) or confirmed severe ROP, given a ROPScore of ≥11 or ≥14.5, respectively. The NPV indicated the probability of not having confirmed ROP (any stage) or severe ROP, given a ROPScore below these thresholds (<11 or <14.5, respectively). Additionally, the accuracy of the ROPScore algorithm was calculated, representing the probability of correct predictions for confirmed ROP (any stage) or severe ROP, using cutoff values of 11 and 14.5, respectively.

### Statistical analysis

The data were processed in an Excel^®^ spreadsheet and analyzed using the IBM SPSS Statistics v.28.0 software. Quantitative variables are presented as mean ± standard deviation (SD). The predictive ability of the algorithms was assessed by calculating sensitivity, specificity, and accuracy values. PPV and NPV were also estimated, factoring the prevalence of ROP in the study population. The normality of the distribution of quantitative variables was assessed using the Kolmogorov--Smirnov test. P-values <0.05 were considered indicative of statistical significance.

## RESULTS

### Clinical characteristics

The mean (±SD) GA and BW in the study population were 28.9 ± 2.1 weeks and 1199 ± 317.2 g, respectively. The mean total duration of oxygen use by any means was 30.1 ± 30.1 days. The mean postmenstrual age at the maximum stage of ROP in preterm infants who developed the disease was 37.4 ± 5.1 weeks (Table 1).

**Table 1:** Quantitative variables of the sample

Variable	N	Mean	Standard deviation
Gestational age (wk)	288	28.9	2.1
Birth weight (g)	288	1199	317.2
Weight 1ª wk (g)	288	1122.8	306.8
Weight 2ª wk (g)	288	1254.9	333.6
Weight 3ª wk (g)	279	1394.1	370.9
Weight 4ª wk (g)	267	1549	390.9
Weight 5ª wk (g)	238	1691.6	424.2
Weight 6ª wk (g)	200	1785	412.3
Time OTI (days)	288	14.8	27.6
Time OTI (days) restricted to cases with OTI	217	19.7	30.3
Duration of oxygen use (days)	288	30.1	30.1
Duration of oxygen use restricted to cases with oxygen use (days)	217	36.3	31.5
Corrected GA in the maximum stage in confirmed ROP cases	110	37.4	5.1

OTI= orotracheal intubation; GA= gestational age; ROP= retinopathy of prematurity; wk= week.

The mean GA and BW of patients who had confirmed severe ROP were lower than those with ROP at any stage, being 26.4 ± 2.2 weeks and 865.5 ± 178.9 g, respectively (Table 2).

**Table 2:** Comparison of cases of ROP (any stage) and cases of confirmed severe ROP in relation to GA and BW

Variable	Confirmed ROP	n	Medium	Standard deviation
Gestational age (weeks)	No	269	29.1	2
	Yes	19	26.4	2.2
Birth weight (g)	No	269	1222.5	311.7
	Yes	19	865.5	178.9

p<0.001; ROP= retinopathy of prematurity; GA= gestational age; BW= birth weight.

### ROPScore and WINROP outcomes

The study revealed notable discrepancies between predicted and confirmed severe ROP cases. Notably, 58.3% of patients received a severe ROPScore classification, and 50.7% triggered a positive alarm sign for severe ROP on WINROP. However, ophthalmologic examinations confirmed severe ROP in only 6.6% (n=19) of the study population. Among those with confirmed severe ROP, treatment modalities included laser therapy (9 patients), anti-VEGF Avastin injections (6 patients), and a combination of laser and Avastin treatment (4 patients).

Among the 288 premature infants studied, 61.8% remained free of ROP throughout. The remaining 38.2% developed ROP, with the following distribution: 14.2% had stage 1; 14.6% had stage 2; 8.3% had stage 3; 0.7% had stage 4; and 0.3% had stage 5. Additionally, plus disease was observed in 4.5% of the infants (Table 3).

**Table 3:** Prevalence of ROP based on ROPScore and WINROP, and confirmed ROP cases

Variable		Total	n	%
ROPScore ≥11	No	288	17	5.9
	Yes		271	94.1
ROPScore ≥14.5	No	288	120	41.7
	Yes		168	58.3
WINROP alarm signal	No	288	142	49.3
	Yes		146	50.7
Confirmed ROP	No	288	178	61.8
	Yes		110	38.2
Confirmed severe ROP	No	288	269	93.4
	Yes		19	6.6
Maximum stage of ROP	0	288	178	61.8
	1		41	14.2
	2		42	14.6
	3		24	8.3
	4		2	0.7
	5		1	0.3
Plus disease	No	288	275	95.5
	Yes		13	4.5

ROP= retinopathy of prematurity.

The average ROPScore in this study was 15.1 ± 2.6 points. For WINROP, the mean corrected GA at alarm signal activation was 30 ± 1.7 weeks (Table 4). Notably, the ROPScore showed 100% sensitivity in predicting confirmed ROP (any stage), using a cutoff point of 11 (Table 5). For predicting severe ROP, ROPScore showed a 100% sensitivity, 44.6% specificity (95% confidence interval [CI] 38.7-50.6), 11.3% PPV (95% CI 6.5-16.1), and a 100% NPV (Table 6).

**Table 4:** Mean ROPScore and mean corrected gestational age of newborns at the time of the WINROP alarm sign

Variable	n	Mean	Standard deviation
ROPScore	288	15.1	2.6
WINROP: Mean GA at positive alarm sign (limited to cases with a positive WINROP alarm sign)	146	30	1.7

**Table 5:** Predictive performance of ROPScore for confirmed ROP (any stage) using cutoff value of 11

	Results %	95% CI
Sensitivity	100	-
Specificity	9.6	5.2-13.9
Accuracy	44.1	38.4-49.8
FP	90.4	86.1-94.8
FN	0	-
PPV	40.6	34.7-46.4
NPV	100	-

CI= confidence interval; FP= false positive; FN= false negative; PPV= positive predictive value; NPV= negative predictive value; ROP= retinopathy of prematurity.

**Table 6:** Predictive performance of ROPScore for confirmed severe ROP using a cutoff value of 14.5

	Results %	95% CI
Sensitivity	100	-
Specificity	44.6	38.7-50.6
Accuracy	48.3	42.5-54
FP	55.4	49.4-61.3
FN	0	-
PPV	11.3	6.5-16.1
NPV	100	-

CI= confidence interval; FP= false positive; FN= false negative; PPV= positive predictive value; NPV= negative predictive value; ROP= retinopathy of prematurity.

The WINROP algorithm showed a 78.9% sensitivity (95% CI 60.6-97.3), 51.3% specificity (95% CI 45.3-57.3), 10.3% PPV (95% CI 5.3-15.2), and 97.2% NPV (95% CI 94.5-99.9) in predicting severe ROP (Table 7).

**Table 7:** Predictive performance of WINROP algorithm for confirmed severe ROP

	Results %	95% CI
Sensitivity	78.9	60.6-97.3
Specificity	51.3	45.3-57.3
Accuracy	53.1	47.4-58.9
FP	48.7	42.7-54.7
FN	21.1	2.7-39.4
PPV	10.3	5.3-15.2
NPV	97.2	94.5-99.9

CI= confidence interval; FP= false positive; FN= false negative; PPV= positive predictive value; NPV= negative predictive value; ROP= retinopathy of prematurity.

## DISCUSSION

The current Brazilian guidelines for ROP screening are based solely on GA and BW^(13)^. Consequently, many preterm infants are included in the screening, with all being considered at equivalent risk for severe ROP development. WINROP and ROPScore algorithms offer enhanced risk stratification by incorporating additional variables. This targeted approach enables screening to focus on high-risk infants. An ideal algorithm to identify preterm infants at risk of severe ROP would have a 100% sensitivity with a reasonable level of specificity^(15)^.

Several studies have demonstrated the effectiveness of the WINROP algorithm as a screening tool. However, its sensitivity varies significantly across different countries and economic contexts. In high-income countries such as Sweden, where the algorithm was developed, and the United States of America, WINROP has demonstrated perfect (100%) sensitivity, identifying all preterm infants with severe ROP^(9^,^10)^. However, middle-income countries such as Mexico have reported lower sensitivity (84%) ^(16)^. A potential explanation for this could be that in the Swedish study, no infant with GA >28 weeks developed stage 3 ROP requiring treatment. In developing countries, infants with higher GA are known to develop ROP more often than in developed high-income countries. These findings suggest that screening criteria should be tailored to the specific population and economic context, taking into account local risk factors and disease patterns^(1^,^3)^.

In the present study, the sensitivity of WINROP (78.9% [95% CI 60.6%-97.3%), was similar to that reported in other middle-income countries such as Mexico. The study identified four premature infants with severe ROP who received treatment, but for whom the algorithm did not trigger an alarm. Notably, these infants had relatively higher GA: 36 weeks, 39 weeks, 43 weeks, and a remarkable 63 weeks. The specificity of the WINROP algorithm in our study was notably lower (51.3%) compared to the original Swedish study (84.5%). This discrepancy resulted in a high rate of false positives and a low PPV (10.3%). Due to this low specificity observed in our study, it would be necessary to generally continue screening for ROP in infants with a positive alarm sign.

The original study that created the ROPScore algorithm obtained a 94% sensitivity and 26% specificity for any stage of ROP. For predicting severe ROP, it showed a 96% sensitivity and 56% specificity. A key advantage of this algorithm lies in its simplicity and practicality, incorporating easily recordable risk factors for ROP, making it suitable for routine use in neonatal intensive care units. Unlike the WINROP algorithm, the ROPScore is recorded only once in a cross-sectional manner^(7)^.

Our study achieved maximum sensitivity in predicting severe ROP, mirroring findings from studies conducted in Brazil and Italy^(17^,^18)^. Notably, ROPScore showed a 100% NPV for both ROP (any stage) and severe ROP, enabling the secure identification of preterm infants not at risk of developing severe ROP. This can inform a decrease in the frequency of ophthalmologic exams and the inclusion of ROPScore in guidelines for ROP screening.

Given the critical importance of detecting every treatable case of ROP, our findings suggest that the WINROP algorithm lacks sufficient sensitivity for use in this population. Ideally, multicenter prospective studies should evaluate the use of WINROP or the appropriateness of its criteria for the Brazilian population.

ROP screening using artificial intelligence (AI) offers a promising solution to address specialist shortages and potential inconsistencies in diagnosis. However, further development is required to ensure that AI-driven ROP screening meets rigorous standards for fairness, generalizability, and bias control^(19)^.

Potential limitations of this study include its single-center scope and retrospective design. More robust prospective studies can provide more definitive evidence.

To conclude, in this study, ROPScore identified all patients at risk for severe ROP. Our findings support the incorporation of ROPScore into Brazilian guidelines to optimize ROP screening and minimize unnecessary ophthalmologic examinations. The suboptimal performance of WINROP in this Brazilian sample highlights the need for country-specific algorithm adjustments.
